# Functional (Dissociative) Narcolepsy: A Case Report and Literature Review

**DOI:** 10.7759/cureus.114725

**Published:** 2026-08-18

**Authors:** Daniel Crail-Meléndez, David Omar López Hernández, Iris E Martínez-Juárez

**Affiliations:** 1 Department of Neuropsychiatry, Unidad de Neuropsiquiatría, Instituto Nacional de Neurología y Neurocirugía Manuel Velasco Suárez, Mexico City, MEX; 2 Department of Medicine, Facultad de Medicina, Universidad Nacional Autónoma de México, México City, MEX; 3 Department of Clinical Neurophysiology, Clínica de Epilepsia, Instituto Nacional de Neurología y Neurocirugía Manuel Velasco Suárez, Mexico City, MEX

**Keywords:** dissociative disorders, fnd, functional cataplexy, functional neurological disorder, narcolepsy-cataplexy, narcolepsy type 1, pseudocataplexy

## Abstract

Functional neurological disorders (FND) are dissociative conditions characterized by an involuntary disruption in the integration of motor, sensory, or cognitive functions, occurring in the absence of structural or biochemical abnormalities. FND can manifest with symptoms that mimic sleep disorders, such as cataplexy - the pathognomonic symptom of narcolepsy type 1 (NT1). NT1 is defined by excessive daytime sleepiness, cataplexy, and orexin deficiency. We report a case of a 20-year-old woman with psychiatric comorbidities who presented with cataplexy-like episodes, hypersomnolence, and hypnagogic hallucinations. She was ultimately diagnosed with FND mimicking narcolepsy, sometimes referred to as “pseudo-narcolepsy.” The diagnosis of FND must be based on the presence of positive clinical signs.

This report reviews the most relevant clinical features supporting the diagnosis of FND in this context. Accurate diagnosis and effective patient communication are essential for therapeutic success, as studies have shown that a clear explanation can significantly reduce symptom frequency and severity.

## Introduction

Functional neurological disorders (FND) are characterized by involuntary motor and sensory symptoms resulting from abnormalities in brain function rather than structural or biochemical pathology. Diagnosis relies on positive clinical features (rule-in) rather than exclusion (rule-out) [1]. The Diagnostic and Statistical Manual of Mental Disorders, Fifth Edition (DSM-5) categorizes FND under somatic symptom disorders, whereas the World Health Organization's International Classification of Diseases (ICD-11) classifies FND under dissociative disorders [2,3]. FND are common, with population studies estimating a prevalence of 50-100 per 100,000 [4,5], accounting for about 5.4% of new outpatient neurology diagnoses and 9% of neurology hospital admissions [4,6].

Narcolepsy is a sleep-wake disorder defined by excessive daytime sleepiness and abnormal rapid eye movement (REM) sleep phenomena [7]. There are the following two subtypes: narcolepsy type 1 (NT1) and narcolepsy type 2. NT1 is distinguished by cataplexy and low cerebrospinal fluid (CSF) orexin (hypocretin) levels, while narcolepsy type 2 lacks cataplexy and typically shows normal orexin concentrations [7].

The prevalence of functional cataplexy remains unknown [8,9], and there are reports of individuals diagnosed with NT1 who also experience episodes of sudden weakness consistent with functional cataplexy [10]. In a case-control study by Menchetti et al., which included 15 patients with “pseudocataplexy,” the authors found that these patients had a lower quality of life compared with individuals with NT1 [8].

Although the ICD-11 includes a residual category for “other specified FND,” neither the ICD-11 nor the DSM-5 recognizes a subtype specifically associated with sleep-wake disorders. This lack of recognition in major classification systems may contribute to underdiagnosis, diminish clinical relevance, or delay appropriate identification.

We describe the case of a young woman who presented with narcolepsy-like episodes with cataplexy and was ultimately diagnosed with FND mimicking narcolepsy. We outlined key clinical features, medical history, and differential diagnoses, particularly those involving functional cataplexy, to support accurate diagnosis.

## Case presentation

A 20-year-old right-handed woman presented to a neuropsychiatry follow-up appointment reporting episodes of sudden sleep accompanied by loss of muscle tone, eyes closed, and lack of response to external stimuli. She remained in this state for approximately 120-300 s, then awoke without confusion and with only residual sleepiness. The episodes did not have a clear trigger, although she sometimes linked them to anxiety. She also reported daytime somnolence and fatigue that interfered with her daily activities.

She experienced sleep paralysis about four times per week, described as a sensation of immobility and dyspnea. Her sleep was nonrestorative. She denied hypnopompic hallucinations, although upon awakening she frequently perceived the sensation that someone was sitting on her bed. She was a single university student whose academic performance had been disrupted by her symptoms.

Her past history was notable for adverse childhood experiences, including physical abuse and sexual abuse during adolescence. At age eight years, she developed motor and vocal tics, which worsened after the abuse. At 16 years, she was diagnosed with Tourette syndrome and major depressive disorder. Severe tics led to four hospitalizations due to self-injury and harm to others. At 18, she was referred to the National Institute of Neurology and Neurosurgery (Mexico City), where functional tics were diagnosed in addition to Tourette syndrome. She denied substance use and had no other significant medical history.

At the end of the consultation, she experienced a typical episode. She suddenly fell asleep with her head flexed over her chest, offered no resistance to passive eyelid opening, and exhibited preserved pupillary light reflexes. Her arms hung loosely at her sides, without strength and without resisting passive movement by the examiner; however, she remained seated throughout the approximately 3-min episode. She gradually regained consciousness and stated that she did not know what had happened, yet she was fully oriented. She was referred to the sleep clinic for evaluation of possible narcolepsy. Her medication regimen included fluoxetine 60 mg/day and olanzapine 10 mg/day.

During a later assessment conducted by the sleep disorders service, the patient experienced another episode. She reported feeling sleepy before suddenly falling asleep while seated, with her eyes closed, lack of facial involvement, and no abnormal movements. Physical examination showed preserved osteotendinous reflexes in all four limbs, normal muscle tone, and intact bilateral pupillary reflexes. Vital signs were within normal limits. At the end of the episode, she reported tiredness and exhibited various combined and alternating motor and vocal tics, which subsided with distraction. Laboratory workup, including metabolic, endocrine, and hematological panels, was unremarkable.

Neurophysiological evaluation

A diagnostic protocol for narcolepsy with cataplexy was initiated according to the criteria of the International Classification of Sleep Disorders-Third Edition, Text Revision (ICSD-3-TR), including polysomnography (PSG) and a multiple sleep latency test (MSLT), along with continued follow-up at the sleep clinic. A nocturnal video-polysomnographic study using a standard montage (including EEG, electrooculogram {EOG}, electromyogram {EMG}, ECG, respiratory plethysmography, and pulse oximetry) revealed mild obstructive sleep apnea/hypopnea syndrome but no evidence of sleep latencies below 8 min. The MSLT showed a mean sleep latency of 16.5 min across four nap opportunities, with no sleep-onset rapid eye movement periods (SOREMPs) observed, failing to meet diagnostic criteria for narcolepsy. She was discharged with recommendations on sleep hygiene and continued follow-up in neuropsychiatry (Table 1).

**Table 1:** Sleep latency values. Summary of the sleep latency values reported. MSLT values are reported in the text. REM: rapid eye movement; MSLT: multiple sleep latency test; N1: non-REM stage 1; N2: non-REM stage 2; N3: non-REM stage 3

Parameters	Patient	Reference value
Sleep onset latency	126 min	<30 min
Latency to N1	2 min	0 min
Latency to N2	20.6 min	10-12 min
Latency to N3 (slow wave sleep)	34 min	20-40 min
REM latency	295 min	60-120 min

Given the absence of PSG/MSLT findings and her history of functional symptoms, she was diagnosed with functional neurological disorder of the narcolepsy type (pseudo-narcolepsy). Notably, after the diagnosis was clearly explained, she no longer experienced further “sleep attacks.”

## Discussion

**Figure 1:**
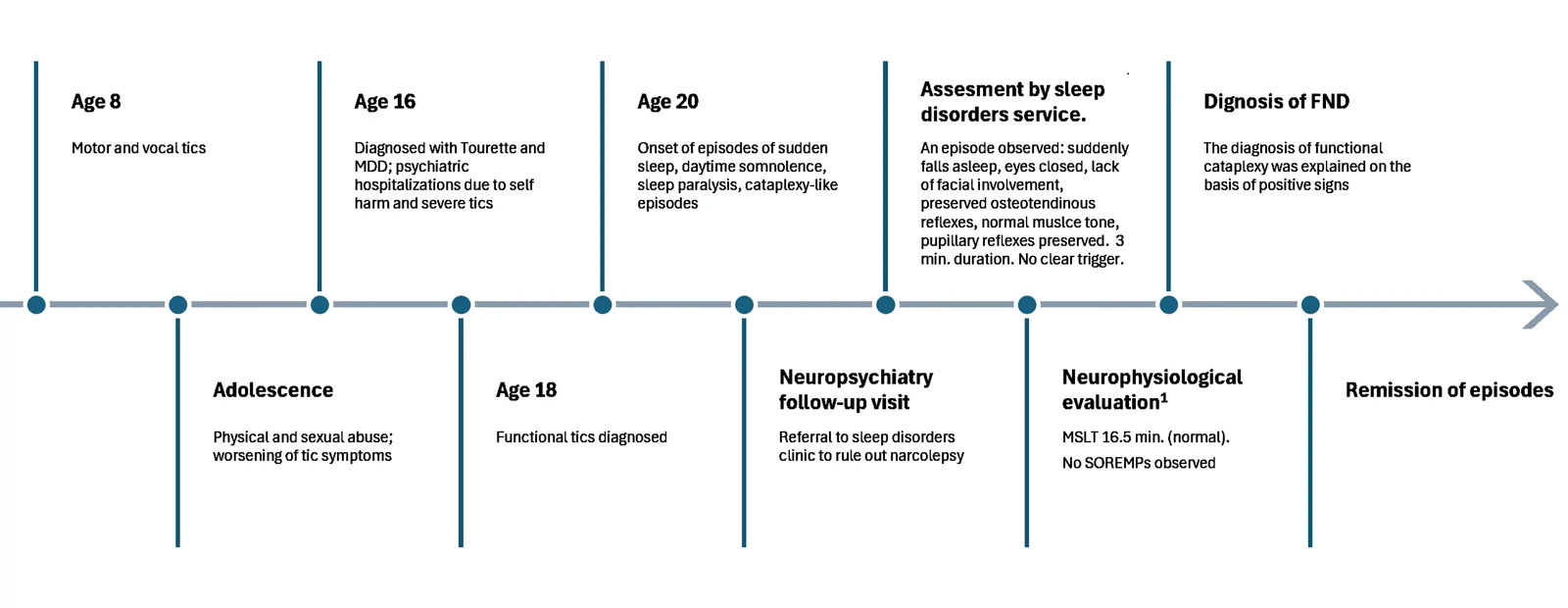
Timeline of symptom progression and diagnostic evaluations. ^1^Neither the criterion of an MSLT of ≤8 min nor that of exhibiting SOREMPs within the first 15 min of falling asleep was met. A timeline is presented, covering the key events leading up to the patient’s assessment and final diagnosis. The ages are presented in years. MDD: major depressive disorder; MSLT: multiple sleep latency test; SOREMPs: sleep-onset rapid eye movement periods; FND: functional neurological disorders

This case meets the diagnostic criteria for FND, as the patient presented with motor and sensory symptoms incompatible with narcolepsy type 1 (NT1). Symptoms impaired her functioning but resolved without pharmacological changes, ruling out the possibility that they were medication side effects and supporting a functional etiology. Figure 1 shows a timeline of the patient’s medical history and the progression of her symptoms leading up to her diagnosis. We did not consider this presentation as FND with isolated cataplexy-like episodes, since the patient not only reported sudden episodes of weakness and sleepiness but also described persistent daytime fatigue, excessive sleepiness, and hypnagogic hallucinations. The mild obstructive sleep apnea/hypopnea syndrome identified in the polysomnography (PSG) could explain some of the daytime hypersomnolence reported by the patient.

Narcolepsy is a rapid eye movement (REM) sleep disorder characterized by hypersomnolence, sleep paralysis, hypnagogic hallucinations, and cataplexy [11]. According to the International Classification of Sleep Disorders-Third Edition, Text Revision (ICSD-3-TR), the diagnosis of NT1 can be established if the criteria listed in Table 2 are met [7].

**Table 2:** Diagnostic criteria for narcolepsy type 1. This table is adapted with permission from the International Classification of Sleep Disorders - Third Edition, Text Revision. Copyright 2023 American Academy of Sleep Medicine [7]. All of criteria A, B, and C must be met to diagnose narcolepsy type 1. SOREMP: sleep-onset rapid eye movement period; MSLT: multiple sleep latency test; PSG: polysomnography

Diagnostic criteria for narcolepsy type 1, orexin/hypocretin deficiency syndrome, narcolepsy with cataplexy
A. Daily periods of irrepressible need to sleep, daytime lapses into drowsiness or sleep
B. At least one of the following elements:
1. Cataplexy and either
a. Mean sleep latency ≤8 min on the MSLT and ≥2 SOREMPs on an MSLT, or
b. A SOREMP (within 15 min of sleep onset) on the nocturnal PSG
2. Low or absent CSF hypocretin‑1 defined as:
a. ≤110 pg/mL, measured by radioimmunoassay, or
b. <1/3 of the normal mean value (using a standardized assay)
C. Symptoms are not better explained by insufficient sleep, circadian rhythm disorders, other sleep disorders, mental disorders, or substance use/withdrawal

It is important to consider that, according to the literature, the clinical presentation, along with the results of polysomnography (PSG) and the multiple sleep latency test (MSLT), constitutes the most relevant diagnostic approach for narcolepsy [12]. In contrast, cerebrospinal fluid (CSF) hypocretin-1 levels and the presence of human leukocyte antigen (HLA)-DQB1 serve only as complementary studies. In the present case, measuring cerebrospinal fluid (CSF) hypocretin levels was deemed unnecessary, given the absence of MSLT findings consistent with narcolepsy and the clinical presentation of cataplexy-like episodes rather than definitive cataplexy.

Cataplexy is the pathognomonic symptom of NT1. It must be differentiated from other conditions that may present with sudden loss of muscle tone, including epilepsy, drop attacks (atonic seizures), and FND with cataplexy-like episodes, commonly referred to as pseudocataplexy. Cataplexy-like events have also been described in hyperkalemic periodic paralysis (Table 3) [8,13-18]. True cataplexy refers to brief episodes of bilateral loss of muscle tone triggered by emotional states without loss of consciousness. Laughter is the most typical trigger (around 50% when tickled).

**Table 3:** Differential diagnosis of cataplexy in adults. ^1^Status cataplecticus can last several minutes. ^2^The most common etiology is vasovagal or neurocardiogenic syncope, usually triggered by orthostatic stress, pain, anxiety, or medical instrumentation. DTRs: deep tendon reflexes

Features	Cataplexy	Functional cataplexy	Syncope	Epilepsy (atonic)
Cause	Loss of hypocretin/hypofunction of the systems regulated by orexins	Dissociation	Cerebral hypoperfusion	Abnormal electrical activity in the brain
Common trigger	Positive emotions (laughing, tickling, surprise)	Mostly negative emotions, atypical or no clear trigger	Depending on etiology^2^: reflex, cardiac, or orthostatic hypotension	Often absent
				Not emotional
Consciousness	Preserved	Preserved or altered	Lost	Altered or absent
DTRs	Abolished	Present	Decreased or transient abolition	Decreased or absent
Duration	Less than 2 min^1^	Long duration	Brief	Brief or ultra-brief
Distribution	Symmetric (most common), focal, or generalized. Facial involvement is typical	Lack of facial involvement. Sometimes asymmetric	Generalized	Axial distribution
Recovery	Fast and complete. Can fall asleep	Variable. May refer tiredness or fatigue	Rapid recovery of consciousness	Variable, postictal state (confusion)
EEG findings	Wake pattern during attack	Normal	Normal	Ictal: burst or polyspikes

Previous reports indicate that patients with NT1 may also experience functional cataplexy episodes [10,19]. As with all functional neurological disorders, functional cataplexy must be diagnosed based on the presence of positive signs. The preservation of tendon reflexes during episodes is a key indicator, along with the absence of hypotonia in the craniocervical region and the abrupt interruption of facial expressions [20,21]. Other suggestive features include atypical or absent triggers, lack of facial involvement (absence of jaw weakness), and prolonged duration (typically >2 min) [8,10,13]. While none of these features is individually pathognomonic for functional cataplexy, their presence in combination strengthens the clinical diagnosis.

The case-control study by Menchetti et al. found that in 90% of the NT1 group, cataplexy spells lasted less than 2 min, and in 53%, less than 10 s. Only 33% of the “pseudocataplexy” group reported laughter as a cataplexy trigger, compared with 100% of the NT1 group [8].

In addition to the differential diagnoses shown in Table 3, it is also important to distinguish these episodes from other functional neurological phenomena. The presence of drowsiness and the brief duration of the events, together with rapid recovery of strength, argue against a functional weakness disorder. The episodes are also inconsistent with functional/dissociative seizures, given the absence of involuntary movements during the events and the patient's description of them as an irresistible state of sleep rather than a state of disconnection [22].

In relation to clinical history, the presence of another type of FND is a strong predisposing factor. Up to 90% of patients diagnosed with FND present with comorbid functional symptoms. The patient described here had previously been diagnosed with a functional neurological disorder characterized by tic-like movements, in conjunction with Tourette syndrome. Comorbidity with a neurological condition is common in functional neurological disorders, and structural abnormalities of the nervous system may predispose a patient to, or trigger, functional symptoms [5].

Among the most consistent neuropathological mechanisms proposed to explain the predisposition to developing different types of functional neurological disorders are limbic system hyperactivity (including amygdala hyperactivity, potentially linked to early-life trauma); aberrant functional connectivity (such as increased connectivity between the salience network, i.e., insula and anterior cingulate cortex and the somatomotor network); and an impaired sense of agency (the feeling that symptoms such as tremors, tics, or weakness are not self-generated), associated with decreased activation or connectivity of the right temporoparietal junction [5,6,23-25].

Additional features that tend to be more prevalent in functional cataplexy, such as psychiatric comorbidities, personality disorders, female predominance, and multiple somatic complaints, are of limited diagnostic utility, as they also occur with relative frequency in true cataplexy [20,21]. Psychological stress and childhood trauma/neglect, as in the present case, are strongly established risk factors, with odds ratios of 3-4 [5].

Currently, no specific treatment protocols exist for FND with cataplexy. Management can draw from approaches to psychogenic nonepileptic seizures (PNES). Symptom remission following clear and respectful communication of the diagnosis, as seen here, is consistent with evidence that patient understanding improves prognosis and, in some cases, may even result in complete remission [22,26]. A retrospective study reported that approximately 38% of patients became free of PNES after being informed of their diagnosis [27]. Psychiatric comorbidity management and addressing psychosocial factors are also essential. Cognitive-behavioral therapy protocols for PNES and other FND may be adapted for functional narcolepsy-like presentations.

## Conclusions

FND presenting with cataplexy-like episodes can be differentiated from true cataplexy in NT1 through positive clinical signs and characteristic features, including preserved reflexes, longer duration, rapid recovery, and the absence of emotion-triggered attacks. As illustrated in this case, patients frequently exhibit comorbid functional symptoms, underscoring the importance of recognizing this overlap to prevent misdiagnosis. A particularly notable aspect of this case is that the patient's “sleep attacks” resolved completely after the diagnosis was clearly explained, without any change in pharmacological treatment. This mirrors findings in the psychogenic nonepileptic seizures (PNES) literature, where diagnostic disclosure and respectful explanation alone can lead to remission in a substantial proportion of patients, highlighting the therapeutic value of clear communication and psychoeducation.

Clinically, accurate identification of functional/dissociative narcolepsy can help avoid unnecessary investigations, such as CSF hypocretin testing or repeat PSG/MSLT studies, and instead guide clinicians toward evidence‑based approaches, including psychoeducation and cognitive-behavioral therapy (CBT)‑informed interventions adapted from PNES protocols. Although this case provides valuable insights, conclusions drawn from a single case report must be interpreted with caution. Future studies are needed to better characterize functional narcolepsy-like presentations, establish standardized diagnostic criteria, and develop tailored treatment strategies. This case supports the notion that functional/dissociative narcolepsy may represent a distinct clinical entity within the spectrum of FND, warranting further characterization and formal recognition.
